# Molecular Identification and Antioxidant Activity Determination among Coffee Varieties Cultivated in Nepal

**DOI:** 10.1155/2023/7744647

**Published:** 2023-11-06

**Authors:** Shreejan Pokharel, Gyanu Raj Pandey, Asmita Shrestha, Rajkumar Shrestha, Dinesh Tiwari, Bignya Chandra Khanal, Sudip Silwal

**Affiliations:** ^1^National Biotechnology Research Center, Nepal Agricultural Research Council, Lalitpur 44700, Nepal; ^2^Shubham Biotech Nepal Pvt. Ltd., Bharatpur-29, Chitwan 44200, Nepal

## Abstract

Coffee is the most popular beverage containing numerous phytochemical components that have antioxidant activity capable of scavenging free radicals. Antioxidant and phenolic contents have considerable benefits for human health. The aim of this study was the molecular identification of 9 coffee samples from the Nepal Agricultural Research Council, Lalitpur, Nepal, and the determination of the antioxidant activity and total phenolic content of green and roasted coffee beans. Molecular identification was performed using ITS-specific PCR followed by sequencing and phylogenetic tree construction using the maximum parsimony method. The DPPH assay was used to determine the antioxidant activity, and the Folin–Ciocalteu (F-C) assay was used to determine the total phenolic content. All the samples belonged to the taxa *Coffea arabica*. The antioxidant activity in roasted beans varied from 2.49 to 4.62 AAE mg/g and from 1.4 to 3.9 AAE mg/g in green beans. The total phenolic content varied from 2.58 to 3.38 GAE mg/g and from 4.16 to 5.36 GAE mg/g for the roasted beans and green beans, respectively. The data revealed that the highest antioxidant content (4.62 AAE mg/g) was found in roasted coffee and that the highest phenolic content (5.36 GAE mg/g) was found in green coffee. The study concludes that roasting increases the antioxidant activity but decreases the phenolic content of coffee.

## 1. Introduction

Coffee is the most popular caffeine-containing beverage worldwide. Coffee belongs to the genus *Coffea* and the family Rubiaceae [1]. *Coffea arabica* (arabica coffee) and *Coffea canephora* (robusta coffee) are the most popular coffee species, comprising 70% and 24% of worldwide commercial production, respectively [2]. *Coffea arabica* is tetraploid (2*n* = 4*x* = 44) and self-fertile, whereas other *Coffea* species are diploid (C*n* = 2*x* = 22) and generally self-incompatible [3].

All coffee species originated from the intertropical forests of Africa and Madagascar [4]. Today, coffee is grown worldwide in tropical and subtropical regions [5]. Coffee is grown in more than 70 countries, among which Brazil is the highest producer with a production of around three million tons in 2019 [6]. The United States, Europe, and Japan are the countries with the highest coffee consumption [7].

In Nepal, coffee was introduced in 1938 A.D. from Myanmar and planted in the Gulmi district. Later in 1968 A.D., Nepal government initiated coffee development in Nepal, brought seeds from India, and distributed them to farmers. The production of coffee is increasing with great potential to provide farm employment and income generation to farmers [8]. More than 30,543 farmers from a 2646 hectare area in the midhills of Nepal, with an altitude of 700–1600 meters above sea level, are actively involved in coffee production [9]. Annually, total coffee production and area of production of coffee in Nepal are increasing by 35% and 28%, respectively [10].

Despite Nepal's long history of coffee farms, few taxonomy investigations have been undertaken. The goal of this study was to identify coffee species and develop a genetic link between them. Many PCR-based molecular marker approaches were employed for genetic diversity assessments, genotype identification in gene bank management, molecular phylogenetic investigations, and species diagnostic procedure creation. These techniques include those based on random amplified polymorphic DNA (RAPD), microsatellites or simple sequence repeats (SSRs), intersimple sequence repeats (ISSRs), amplified fragment length polymorphism (AFLPs), and internal transcribed spacers (ITS) sequences [11].

Among various methods, DNA barcoding using internal transcribed spacers (ITSs) was more efficient in the molecular identification of coffee [12]. The internal transcribed spacer (ITS) region of the nuclear ribosomal cistron consists of two spacers, ITS1 and ITS2, which are located between the small subunit 18S ribosomal gene and the large subunit 26S ribosomal gene and are separated by the 5.8S gene. Characteristics such as biparental inheritance, simplicity, intragenomic homogeneity, intergenomic diversity, and minimal functional constraint have led ITS-based phylogenetic analysis to dominate the plant molecular phylogenetic approach. Along with these advantages, there is the presence of a large body of sequence data for this region; recent studies have used the ITS region to study the phylogenetic relationship among several plant genera [13, 14].

Coffee is a rich source of dietary antioxidants, and this property, combined with the fact that coffee is one of the world's most popular beverages, has led to the consideration that coffee is a major contributor to dietary antioxidant intake and is regarded as a functional food [15]. Coffee is a complex food matrix with numerous phytochemical components that have antioxidant activity capable of scavenging free radicals. The most important bioactive compounds include phenolic compounds (chlorogenic acids and derivatives), methylxanthines (caffeine, theophylline, and theobromine), diterpenes (cafestol and kahweol), nicotinic acid (vitamin B3), and its precursor trigonelline, magnesium, and potassium [16].

Antioxidants are micronutrients that have gained importance in recent years because of their ability to neutralize free radicals [17]. Although coffee is consumed primarily because of its pleasant flavor and stimulating properties, more recent investigations have indicated potential health benefits associated with the beverage, including a reduced incidence of several chronic and degenerative diseases, such as cancer, cardiovascular disorders, diabetes, and Parkinson's disease, and reduced mortality risks [18, 19]. Some studies have shown that coffee components can trigger tissue antioxidant gene expression and protect against gastrointestinal oxidative stress [20].

Since the introduction of coffee in Nepal, only the socioeconomic aspect of coffee has been studied, but no major work on its genetic identification and diversity has been initiated. There are few research studies on its characterization which lay a foundation but do not give complete information. The present work aims to evaluate the phylogenetic relationship, antioxidant content, and total phenolic content of 9 coffee samples grown in Nepal. Because coffee is the most popular beverage, the presence of antioxidant activity and phenolic contents is highly beneficial to coffee users.

## 2. Materials and Methods

### 2.1. Sample Collection

Green coffee beans were obtained to access the antioxidant property, and young coffee leaves were used for molecular identification. Samples as shown in Table 1 were collected from the Nepal Agricultural Research Council, Lalitpur, Nepal (latitude: 27.6550983612, longitude: 85.3270125389), and stored in plastic bags with zippers.

### 2.2. Identification of the Selected Coffee Sample

(1) Sample preparation: The leaves were cleaned and stored at −80°C for DNA extraction.
(2) DNA extraction and sequencing: DNA was extracted from leaf samples using the Doyle and Doyle method with some optimizations [21]. The ITS2 region of genomic DNA was amplified using primers ITSL (5′ TCGTAACAAGGTTTCCGTAGGTG 3′) and ITSR (5′ TATGCTTAAAYTCAGCGGG 3′) [22]. The PCR product was sequenced at the sequencing facility of Macrogen Inc., South Korea. Raw sequences were obtained from Macrogen. Raw sequences were assembled and trimmed using Codon Code Aligner. The generated contig sequences were subjected to BLASTN, and the database “Standard databases (nr, etc.)” was selected. Highly similar sequences were obtained in the FASTA format for phylogenetic analysis.
(3) Maximum parsimony analysis of taxa: The entire ITS region of coffee leaf samples was sequenced, and sequencing results were analyzed to construct a phylogenetic tree using the maximum parsimony method for the validation of the molecular identity of 9 coffee samples [23]. The bootstrap consensus tree inferred from 2000 replicates is used to represent the evolutionary history of the taxa analyzed [24]. This analysis involved 20 nucleotide sequences. All positions with less than 95% site coverage were eliminated; i.e., fewer than 5% alignment gaps, missing data, and ambiguous bases were allowed at any position (partial deletion option). Evolutionary analyses were conducted using MEGA X [25]. Genetic distances among the sample sequences were used to interpret the similarities between the species. Phylogenetic analysis was used to confirm the molecular identity of coffee samples by comparing the results of nucleotide sequence alignments with those of standard sequences such as *C. arabica, C. canephora,* and *C. congensis.*
(4) Accession numbers of the nucleotide sequences used are as follows:
  - Accession numbers of the samples used in our study:
  - OP159451.1, OP159452.1, OP159453.1, OP159454.1, OP159455.1, OP159456.1, OP159457.1, OP159458.1, OP159459.1
  - Accession numbers from the NCBI database:
  - MN719947.1, MN719952.1, MZ734346.1, MN734347.1, MK611791.1, MK611792.1, MK615729.1, MK615731.1, MZ734331.1, MZ734348.1, AF542983.1

### 2.3. Methods for Measuring Antioxidant Properties

(1) Sample preparation: The obtained green beans were roasted at 200°C for 20 min. Both green and roasted coffee beans were ground to powder in a standard coffee grinder. One gram of each coffee sample (both green and roasted) was resuspended in 100 mL of hot water (50–60°C) for 3 min. The coffee brews obtained were then filtered using Whatman Filter Paper No. 43. Finally, the coffee extracts were used for analysis, and the remaining extracts were stored at a temperature of −20°C for future use.
(2) DPPH assay: The antioxidant activity of coffee samples was estimated according to the procedure reported by Morales and Jimenez-Perez [26] with a few modifications. A solution of 74 mg/L DPPH in methanol was prepared fresh for the assay. 20 *μ*L aliquot of the sample was added to 1 mL of DPPH, shaken, and incubated for 30 min in the darkroom at room temperature. Absorption was measured at 520 nm using a UV-Vis spectrophotometer. The reaction mixture containing control (1 mL methanol) and reference standard (1 mL DPPH) was also measured. The measurement was compared with a calibration curve prepared with ascorbic acid at various concentrations (0.01–0.4 mg/mL) [27]. The results were expressed in AAE mg/g using the standard curve equation:
  (1) $$\begin{array}{c} y=-1.485x+1.594,R^{2}=0.991, \end{array}$$
where *y* is the DPPH absorbance at 520 nm and *x* is the ascorbic acid concentration in different samples expressed in mg/ml, which is then expressed as AAE mg/g of dry coffee powder.
(3) Assessment of total phenolic content (TPC) of coffee grounds: The TPC of coffee grounds was determined by the spectrophotometric method using Folin–Ciocalteu (F-C) reagent [28]. A 20 *μ*L sample was added to a tube containing 1 mL deionized water, followed by the addition of 100 *μ*L Folin–Ciocalteu (1 : 10) reagent. The reaction mixture was incubated for 3 min at room temperature. Then, 280 *μ*L of 25% w/v sodium carbonate solution and 600 *μ*L deionized water were added to the reaction mixture and mixed properly. After 1 h of incubation at room temperature in the dark, absorbance was measured at 765 nm using a UV-Vis spectrophotometer against the blank solution (F-C reagent and water only). The measurement was compared to a calibration curve prepared with gallic acid solution at a concentration range (0.0005–0.5), and the total phenolic content was expressed as GAE mg/g using the standard curve equation:
  (2) $$\begin{array}{c} y=53.5443x+0.0089,R^{2}=0.997, \end{array}$$
where *y* is the absorbance at 765 nm and *x* is the total phenolic content in the different samples expressed in mg/mL, which is then expressed as GAE mg/g of dry coffee powder.

### 2.4. Statistical Analysis

Data analysis was performed using OriginPro 9.0 and Microsoft Excel. ANOVA was performed for quantitative data, and the Tukey test was used to compare the means at a 95% confidence interval.

## 3. Result and Discussion

The entire ITS region of coffee leaf samples was sequenced, and sequencing results were analyzed to construct a phylogenetic tree using the maximum parsimony method to validate the molecular identity of nine coffee samples. Genetic distances among the sample sequences were used to interpret the similarities between the species. Phylogenetic analysis was used to confirm the molecular identity of coffee samples by comparing the results of nucleotide sequence alignments with those of standard sequences such as *C. arabica, C. canephora,* and *C. congensis.*

The pairwise nucleotide-sequence divergence (Jukes–Cantor model) among the coffee taxa ranged from 0% to 0.9% within different taxa of *C. arabica*, as shown in Table 2. A divergence of 0.9% was detected between Syangja_Special and NARC-C1. NARC-C4 and NARC-C1 were 0.75% divergent from each other. NARC-C4, NARC-C3, NARC-C2, and Gulmi_Local were detected with 0.6% divergence from Syangja_Special. All samples, except Syangja_Special, were detected with no divergence with NARC-C5. These divergences were attributable to deletion and insertion events, and gaps were introduced to align the sequences that appeared particularly important within the different coffee species [4].

**Figure 1:**
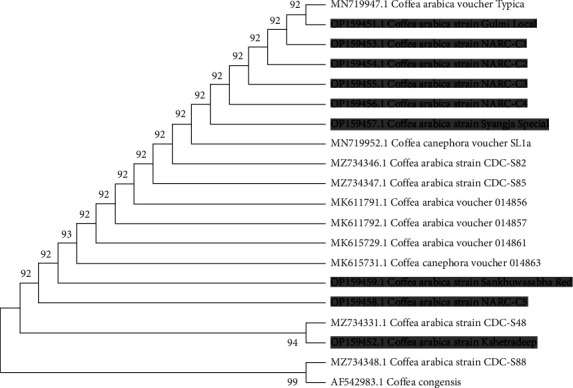
Maximum parsimony tree generated from internal transcribed spacer (ITS) sequence data. Branches corresponding to partitions reproduced in less than 50% of bootstrap replicates are collapsed. The percentage of replicate trees in which the associated taxa clustered together in the bootstrap test is shown next to the branches.

Maximum parsimony (MP) analyses resulted in a single, fully resolved tree with a length of 242, a consistency index of 1.0000, and a retention index of 1.0000. The maximum parsimony tree (Figure 1) shows that all the samples in this study were *C. arabica*. The most effective method for DNA barcoding of plants involves the integration of both coding and noncoding genetic markers. Most of the research on barcoding uses conserved regions such as rbcL and matK regions which show great variability. Regardless, ITS is considered a standard barcode for most of the plant and some algal species [30].

As the molecular identification of Nepali coffee genotypes was unknown, the genotypes were cultivated solely on verbal information. Very few studies have been undertaken in Nepal for the identification of coffee species. Most of the studies were based on socioeconomic aspects, and there was no sufficient information regarding its identification and diversification. Previously, a phylogenetic analysis of coffee samples from Gulmi (Coffee Development Center) was undertaken, which showed that all the samples were *Coffea arabica* [12]. These studies provide valuable information regarding the status of coffee genotypes for farming and varietal improvement. It acts as a starter for identification studies of coffee found in Nepal, with a prominent aspect of researching for better quality output for the farmers of Nepal.

Homogenization mechanisms linked with concerted evolution are thought to maintain overall sequence homogeneity among members of a gene family, such as nuclear rDNA [31]. As a result, rDNA repeats are usually very similar within individuals and species, although differences may accumulate between species, as we observed in pairwise comparison [32]. The observed deficiency in the homogenization mechanisms may be related to the long-life cycles of coffee trees, in the same manner, that nucleotide substitution rates have been reported to be related to the length of the reproductive cycle [5, 33]. Moreover, it is most likely that spontaneous interspecific hybridization occurs between taxa and is involved in speciation. Given their important role in posttranslational processing, ITS regions are considered to be quite conserved [34]. However, ITS phylogeny was successful in distinguishing the coffee species in our investigation. Further use of other phylogeny techniques must be carried out to identify and validate the species level of genotypes. Nevertheless, this discovery lends credibility to the use of ITS as a phylogenetic reconstruction technique for intraspecies differentiation in coffee species.

**Figure 2:**
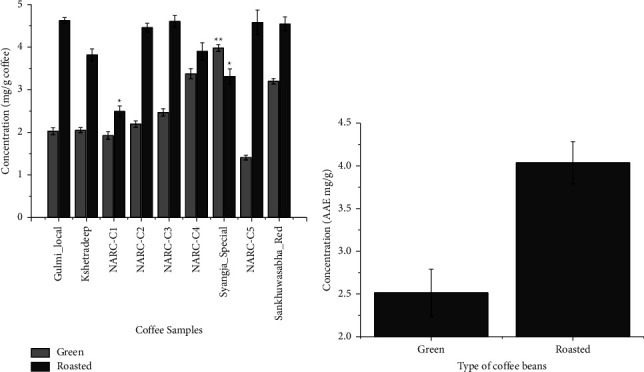
Antioxidant activity was assessed by the DPPH assay. (a) Graph showing the concentration of ascorbic acid equivalent compounds (mg/g coffee) of different coffee samples (for both green and roasted beans). ^*∗*^The concentration of ascorbic acid equivalent compounds (roasted samples) is significantly lower than the mean value of Gulmi_local at *α* = 0.05. ^*∗∗*^The concentration of the ascorbic acid equivalent compound (green sample) of Syangja_Special is significantly higher than the mean value of other samples at *α* = 0.05. (b) Graph expressed as the mean concentration of antioxidant activity of green and roasted coffee beans expressed in AAE mg/g.

The antioxidant activity expressed as milligrams of ascorbic acid equivalent antioxidant capacity per gram coffee extract (AAE mg/g) was determined for 9 coffee samples (green and roasted). The antioxidant activity in roasted beans varied from 2.49 to 4.62 AAE mg/g and from 1.4 to 3.9 AAE mg/g in green beans as shown in Figure 2(a). The concentration of ascorbic acid equivalent compounds was highest in a roasted sample (Gulmi_Local) and lowest in a green coffee bean (NARC-C5) at a confidence interval of *p* < 0.05. The average value ranged from 2.5 to 4.0 AAE mg/g for green and roasted coffee beans as shown in Figure 2(b). A similar study found that the Trolox equivalent antioxidant capacity varied from 0.88 to 1.29 TEAC mg/g and from 0.08 to 1.83 TEAC mg/g in green and roasted coffee samples, respectively [35].

The results suggest that green beans possess antioxidant activity, but the activity is significantly higher in roasted beans. Green coffee beans are rich in bioactive compounds such as caffeine, trigonelline, chlorogenic acids, tocopherols (*α*, *β*, *γ*), and diterpenes (mainly kahweol and cafestol) with antioxidant properties [36]. Dark-roasted coffee has a significantly low antioxidant activity, whereas light-roasted coffee has the highest antioxidant activity [37, 38]. A study showing the effect of roasting conditions on the antioxidant activity of Colombian coffee illustrates that roasted beans exhibit a significantly higher antioxidant activity than unroasted beans, which may be due to the Maillard reaction and the release of bound polyphenols from plant cells [39]. Because antioxidant compounds provide health benefits, coffee as a popular beverage will be of great interest to those trying to increase their intake of these nutrients.

**Figure 3:**
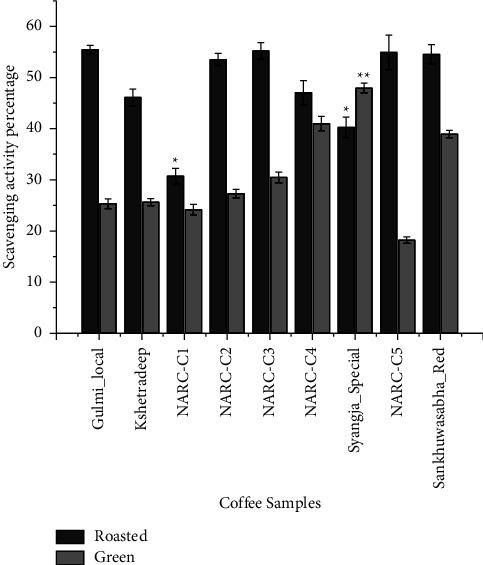
Graph showing the comparison of the free radical scavenging activity percentage of coffee samples. ^*∗*^Free radical scavenging activity percentage (roasted samples) is significantly lower than the mean value of Gulmi_local at *α* = 0.05. ^*∗∗*^Free radical scavenging activity percentage (green samples) is significantly higher than the mean value of other samples at *α* = 0.05.

Among the nine coffee samples, eight roasted samples exhibited an increased antioxidant activity compared with their respective green beans, whereas in sample Syangja_Special, the opposite was observed, as shown in Figure 3, and specifically, in varieties Gulmi_Local, Kshetradeep, NARC-C2, NARC-C3, and NARC-C5, the roasted beans exhibited a significant increase in antioxidant activity compared with the green beans by 30.13%, 20.45%, 24.76%, and 36.74%, respectively. However, in Syangja_Special, the antioxidant activity decreased by 7.69% after roasting. In a similar study, roasted beans showed a significantly (*p* < 0.01) higher antioxidant activity than green beans by 24.1%–27.9% [28]. High temperatures employed during roasting are well recognized to have a significant impact on the chemical makeup of coffee beans [33, 34]. During roasting, some substances, such as chlorogenic acids, are broken down, whereas other compounds, such as melanoidins, are composed [10, 35].

**Figure 4:**
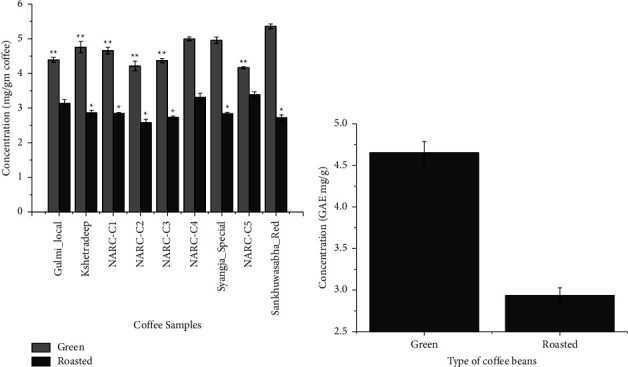
(a) Graph showing the comparison of gallic acid equivalent phenolic compounds in coffee samples. ^*∗*^The gallic acid equivalent phenolic compound (roasted samples) is significantly lower than the mean value of NARC-C4 at *α* = 0.05. ^*∗∗*^The gallic acid equivalent phenolic compound (green samples) is significantly lower than the mean value of Sankhuwasabha_Red at *α* = 0.05. (b) Graph expressed as the mean concentration of phenolic compounds of green and roasted coffee beans expressed in GAE mg/g.

Total phenolic content (TPC) was expressed as milligrams of gallic acid equivalent compounds per gram of coffee extract (GAE mg/g). Phenolic compounds were found to be significantly higher in unroasted samples than in roasted samples.  Figure 4(a) illustrates that the highest TPC was found in a green sample (Sankhuwasabha_Red) and the lowest in a roasted sample (NARC-C2) at a confidence interval of *p* < 0.05. TPC varied from 2.58 to 3.38 mg GAE/g and from 4.16 to 5.36 mg GAE/g for the roasted beans and green beans, respectively. The average value ranged from 2.93 to 4.65 GAE mg/g as shown in Figure 4(b). Our findings were remarkably comparable to those of a study that found that the extractable fractions of green and roasted coffee beans had TPCs that ranged from 114.71 to 172.49 and 92.03 to 134.70 mg of GAE per 100 grams, respectively [40]. All green coffee samples possessed a higher number of phenolic compounds than roasted beans. This is because the total phenolic content and antioxidant activity of coffee beans are significantly influenced by the roasting levels and geographic origin. During roasting, phenolic compounds are partially degraded and/or bound to polymer structures depending on roasting conditions [29]. The low concentration of polyphenols obtained in the present study is in agreement with those presented in the relevant literature, even though, depending on the variety, large variations have been detected [36, 41, 42]. The lower concentration of polyphenol could be explained by the fact that phenolic compounds are often more soluble in alcohol extracts than water, which was used in this study [35].

The results showed that the samples were *C. arabica*. According to various studies, arabica coffee has a higher antioxidant activity and total phenolic content [43, 44]. From this perspective, considering the high antioxidant activity and phenolic content, consumption of these coffees has potential health benefits.

## 4. Conclusion

All of the samples in our investigation were *Coffea arabica,* with little or no variation within the species. In this study, the concentration of compounds exhibiting antioxidant activity in roasted coffee beans was greater than that in green beans. The highest concentration of ascorbic acid equivalent compounds was found in Gulmi_Local among the roasted beans and Syangja_Special among the green beans. Green coffee beans possessed a higher number of phenolic compounds than roasted beans. The total phenolic content was found to be highest in NARC-C4 among the roasted samples and Sankhuwasabha_Red among the green samples. Thus, roasting affects the chemical makeup of coffee beans by generating and breaking down compounds and affecting the antioxidant activity and phenolic contents of coffee beans.

These findings help us understand the genetic relationships between numerous coffee varieties in Nepal. Based on these findings, further studies might examine the genetic diversity of coffee varieties in Nepal and perhaps enhance the region's coffee-growing methods.

## Figures and Tables

**Table 1:** Descriptions of 9 coffee samples used in this study.

Lab code	Field code	Sample type	Source (location)	Young leaf color	Fruit color
Sankhuwasabha_Red	Sankhuwasabha local	Beans/leaves	Sankhuwasabha	Green	Maroon red (G to R)
Syangja_Special	Syangja special	Beans/leaves	Highland coffee nursery, Syangja	Green	Red (G to R)
NARC-C5	Bourbon Vermello	Beans/leaves	HRS, Malepatan, Pokhara	Light green	Red (G to R)
NARC-C4	Catisic	Beans/leaves	—	—	—
NARC-C3	Pacas	Beans/leaves	HRS, Malepatan, Pokhara	Green	Red (G to R)
NARC-C2	Catimor	Beans/leaves	HRS, Malepatan, Pokhara	Green	Red (G to R)
NARC-C1	Selection 10	Beans/leaves	HRS, Malepatan, Pokhara	Green	Maroon red (G to R)
Kshetradeep	Chhetradeep	Beans/leaves	HRS, Malepatan, Pokhara	Green	Red (G to R)
Gulmi_Local	Gulmi local	Beans/leaves	CDC, Gulmi	Green	Red (G to R)

G = green; R = red. Data received from the Nepal Agriculture Research Center, NARC.

**Table 2:** Pairwise comparisons of ITS2 sequences obtained from the nine coffee trees listed in Table 1.

SN		1	2	3	4	5	6	7	8	9
1	Sankhuwasabha_Red									
2	NARC-C5	0.0000								
3	Syangja_Special	0.0046	0.0031							
4	NARC-C4	0.0015	0.0000	0.0060						
5	NARC-C3	0.0015	0.0000	0.0060	0.0015					
6	NARC-C2	0.0015	0.0000	0.0060	0.0015	0.0000				
7	NARC-C1	0.0015	0.0000	0.0090	0.0075	0.0000	0.0000			
8	Kshetradeep	0.0000	0.0000	0.0046	0.0015	0.0030	0.0030	0.0030		
9	Gulmi_Local	0.0015	0.0000	0.0060	0.0015	0.0000	0.0000	0.0000	0.0030	

The number of base substitutions per site between sequences is shown. Analyses were conducted using the Jukes–Cantor model [29]. The rate variation among sites was modeled using a gamma distribution (shape parameter = 1).

## Data Availability

The data will be available from corresponding author upon request.
